# Active Tuberculosis Incidence and Characteristics in Patients Treated with Tumor Necrosis Factor Antagonists According to Latent Tuberculosis Infection

**DOI:** 10.1038/s41598-017-06899-1

**Published:** 2017-07-25

**Authors:** Eun Hye Lee, Young Ae Kang, Ah Young Leem, Moo Suk Park, Young Sam Kim, Se Kyu Kim, Joon Chang, Song Yee Kim

**Affiliations:** Division of Pulmonology, Department of Internal Medicine, Institute of Chest Diseases, Severance Hospital, Yonsei University College of Medicine, Seoul, Republic of Korea

## Abstract

This study aimed to determine the incidence and characteristics of active tuberculosis (TB) in patients treated with tumor necrosis factor (TNF) antagonists according to baseline latent tuberculosis infection (LTBI). Data were retrospectively obtained from 702 patients aged ≥20 years treated with TNF antagonists between November 2005 and June 2016 at Severance Hospital, a tertiary referral hospital in Seoul, South Korea. The interferon-gamma release assay (IGRA) with or without a tuberculin skin test (TST) was used to diagnose LTBI. Of the total of 702 patients, LTBI was diagnosed in 255 (36.3%) patients. 23.9% (168/702) had positive IGRA results, and 32.2% (165/512) had positive TST results. Five patients developed active TB after LTBI treatment, and 6 developed TB despite baseline negative LTBI results. After adjustment for age and sex, the standardized TB incidence ratio was 6.01 (95% CI 1.94–14.04) in the LTBI group and 5.14 (95% CI 1.88–11.18) in the baseline negative LTBI group. Clinicians should be aware of the risk of active TB in patients treated with TNF antagonists despite baseline negative LTBI screening results. Regular monitoring and serial tests should be considered during long-term TNF antagonist therapy, especially in intermediate to high TB burden country.

## Introduction

Tumor necrosis factor (TNF) and TNF receptors play important roles in mediating the immune system and inflammatory systems. Recently, TNF antagonists have been increasingly used in the treatment of chronic inflammatory diseases such as rheumatoid arthritis (RA), ankylosing spondylitis (AS), psoriasis, and inflammatory bowel disease. Despite the effectiveness of TNF antagonist treatment, one of the most serious side effects is an associated increased risk of developing tuberculosis (TB), mostly through reactivation of latent tuberculosis infection (LTBI)^1–3^. Most guidelines suggest that LTBI should be screened for before the initiation of TNF antagonist therapy, and recommend LTBI treatment^2, 4–7^. However, screening strategies and treatment regimens differ between countries. Despite rapid economic growth and urbanization, South Korea has been ranked as having the highest incidence and prevalence of TB among Organization for Economic Cooperation and Development (OECD) member countries. The annual incidence of notified active TB cases is approximately 78 per 100,000 in the general population in 2011, and the estimated prevalence of latent TB is 33%^8, 9^. Therefore, the risk of developing TB through LTBI reactivation or a new infection in patients undergoing TNF antagonist therapy in Korea is reportedly higher than it is in countries with a low TB burden^10, 11^. Studies investigating the incidence and characteristics of active TB in patients undergoing TNF antagonist therapy according to baseline LTBI are important in countries with a relatively high TB burden. In this study, we evaluated baseline LTBI screening results in patients treated with TNF antagonists in a 2000-bed tertiary referral hospital in Seoul, South Korea, and determined the incidence and characteristics of active TB according to baseline LTBI status.

## Patients and Methods

### Study population

**Figure 1:**
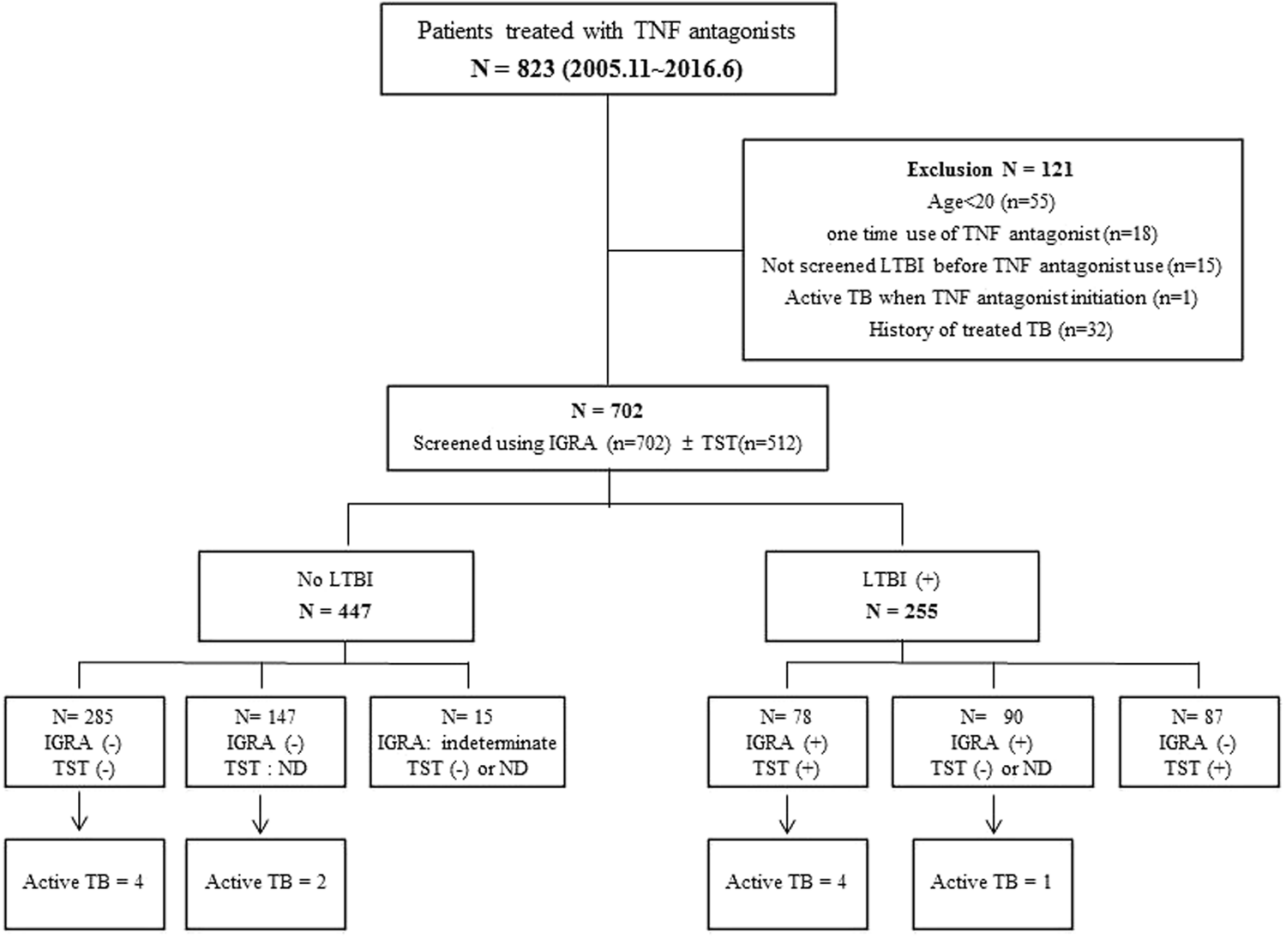
LTBI screening and active TB development in patients treated with TNF antagonists. TNF, tumor necrosis factor; TB, tuberculosis; LTBI, latent tuberculosis infection; IGRA, interferon-gamma release assay; TST, tuberculin skin test; ND, not done.

A total of 823 patients treated with TNF antagonists between November 2005 and June 2016 at Severance Hospital, a 2000-bed tertiary referral hospital in Seoul, South Korea were analyzed. Medical records were retrospectively reviewed. Patients who were under 20 years of age (*n* = 55), only used TNF antagonists once (*n* = 18), were not properly screened for LTBI before TNF antagonist therapy (*n* = 15), and those with a history of active TB who had had proper anti-TB treatment (*n* = 32) or an active tuberculosis diagnosis at the time of TNF antagonist initiation (*n* = 1) were excluded (Fig. 1).

### Diagnosis and treatment of LTBI

The interferon gamma (IFN-ɣ) release assay (IGRA) using QuantiFERON^®^ TB-Gold In Tube (QFT-GIT) with or without a tuberculin skin test (TST) was performed in a total of 702 patients to screen for LTBI before TNF antagonist therapy in accordance with the Korean guidelines^4^. Chest radiographs and symptoms were evaluated to exclude active TB. The QFT-GIT was performed in accordance with the manufacturer’s instructions (Cellestis Ltd., Carnegie, Victoria, Australia). A positive QFT-GIT result was defined as an IFN-ɣ response to TB antigen minus that of the nil tube of ≥0.35 IU/mL. The TST was performed in 515 patients. A 2-TU dose of purified protein derivative RT-23 (Statens Serum Institute, Copenhagen, Denmark) was injected intradermally into the forearm, in accordance with the Mantoux method^12^. A positive TST result was defined as an induration 10 mm in transverse diameter after 48–72 h^4^. Where IGRA or TST results were positive, patients were diagnosed as LTBI and patients were treated with either 9 months of daily isoniazid (9H) or 3 months of daily isoniazid plus rifampicin (3HR) according to the treating physician’s preference. Treatment completion was defined as the ingestion of >80% of all prescribed medications within 43 weeks for 9H or within 16 weeks for 3HR^13^. Previous healed TB in chest radiography was defined by the presence of apical fibronodular lesions, calcified lymph nodes, calcified solitary nodules, and pleural thickening^14^.

### Follow-up

Follow-up duration was calculated until the development of active TB, death, or transfer to another hospital. Otherwise, patients were observed until September 2016, with a focus on active TB development after TNF antagonist therapy.

### Calculation of TB incidence

Standardized incidence ratio (SIR) adjusted for age and sex was estimated using data from the general population of Korea, obtained from the Korean National Statistics Office^15^. The SIR was calculated as the ratio of the observed number of active TB cases among patients receiving TNF antagonists to the expected number of active TB cases with a 95% confidence interval (CI). The expected number of active TB cases treated with TNF antagonists was extrapolated from the active TB incidence in the general population in 2011 (the midpoint of 2005–2016), which was provided by the Korean Centers for Disease Control and Prevention^9^.

### Statistical analysis

Continuous data were expressed as mean ± SD or median and range. Percentages and frequencies were used for categorical variables. Categorical variables were compared using chi-square tests or Fisher’s exact test, and Student’s *t-*tests were performed for continuous variables. *p* values < 0.05 were considered significant. All statistical analyses were performed using SPSS version 18.0 (SPSS Inc. Chicago, IL, USA).

### Ethics

The research protocol was approved by the Institutional Review Board (IRB) of Severance Hospital (IRB No. 4-2016-0989). The need for informed consent was waived due to the retrospective nature of the study.

## Results

### Baseline characteristics

Table 1 shows the clinical characteristics of patients treated with TNF antagonists. Of the total of 702 patients, 255 were diagnosed with LTBI (the LTBI group) before TNF antagonist use, and 447 tested negative for LTBI (the no-LTBI group). The median age of the 702 patients was 44 years (range 20–84) and the LTBI group was significantly older than no-LTBI group (mean 50 years *vs*. mean 39 years, *P* < 0.001). There were 356 (50.7%) male patients.

**Table 1:** Baseline characteristics of patients who were treated with TNF antagonists.

Characteristic	LTBI (n = 255)	No LTBI (n = 447)	Total (n = 702)	*P*
Age, years, median (range)	50 (21–78)	39 (20–84)	44 (20–84)	<0.001
Sex (male), n (%)	134 (52.5)	222 (49.7)	356 (50.7)	0.462
BMI, mean ± SD, kg/m^2^	22.83 ± 3.41	21.76 ± 3.93	22.19 ± 3.76	<0.001
Chest x-ray finding suggestive healed TB, n (%)	9 (3.5)	2 (0.4)	11 (1.6)	0.003*
Main diagnosis, n(%)				
Rheumatoid arthritis	119 (46.7)	140 (31.3)	259 (36.9)	<0.001
Ankylosing spondylitis	81 (31.8)	93 (20.8)	174 (24.8)	0.001
Crohn’s disease	16 (6.3)	125 (28.0)	141 (20.1)	<0.001
Ulcerative colitis	24 (9.4)	56 (12.5)	80 (11.4)	0.211
Others	15 (5.9)	33 (7.4)	48 (6.8)	0.449
Comorbidity, n (%)				
HTN	30 (11.8)	46 (10.3)	76 (10.8)	0.546
DM	19 (7.5)	19 (4.3)	38 (5.4)	0.072
Chronic lung disease^†^	3 (1.2)	10 (2.2)	13 (1.9)	0.394*
Chronic kidney disease	6 (2.4)	6 (1.3)	12 (1.7)	0.369*
Chronic liver disease	3 (1.2)	5 (1.1)	8 (1.1)	1.000*
Malignancy	10 (3.9)	10 (2.2)	20 (2.8)	0.197
TNF antagonist, n (%)				
Infliximab	47 (18.4)	161 (36.0)	208 (29.6)	<0.001
Adalimumab	57 (22.4)	139 (31.1)	196 (27.9)	0.013
Golimumab	20 (7.8)	31 (6.9)	51 (7.3)	0.656
Etanercept	48 (18.8)	34 (7.6)	82 (11.7)	<0.001
Two or more TNF antagonists	83 (32.5)	82 (18.3)	165 (23.5)	<0.001
Immunosuppressive drugs, n (%)				
Steroid	146 (57.3)	201 (45.0)	347 (49.4)	0.002
Antimetabolite^‡^	149 (58.4)	292 (65.3)	441 (62.8)	0.069
Calcineurin inhibitor^§^	13 (5.1)	7 (1.6)	20 (2.8)	0.007
Combination	172 (67.5)	309 (69.1)	481 (68.5)	0.646
Other DMARDs^¶^	136 (53.3)	274 (61.3)	410 (58.4)	0.039
None	5 (2.0)	19 (4.3)	24 (3.4)	0.108
Duration of TNF antagonist use, median (range), months	18.6 (0.5–114.8)	12.7 (0.5–108.7)	15.5 (0.5–114.8)	0.002
Duration of follow up, median (range), months	32.6 (1.1–131.3)	31.8 (0.5–126.7)	32.1 (0.5–131.3)	0.007
Results of LTBI test				
TST induration (mm; median, IQR)	12 (8.75–15)	0 (0–0)	0 (0–11)	<0.001
IFN- γ concentration (IU/mL;median, IQR)	0.74 (0.08–2.97)	0.01 (0–0.04)	0.02 (0–0.29)	<0.001

Data are presented as numbers (percentages) unless otherwise indicated. *Compared using Fisher’s exact test. ^†^Chronic obstructive pulmonary disease, asthma, bronchiectasis, interstitial lung disease. ^‡^Methotrexate, Azathioprine. ^§^Cyclosporine, Tacrolimus. ^¶^5-Amino salicylic acid derivative, Leflunomide. LTBI, latent tuberculosis infection; BMI, body mass index; TB, tuberculosis; HTN, hypertension; DM, diabetes mellitus; TNF, tumor necrosis factor; DMARD, disease modifying anti-rheumatic drugs.

Eleven patients exhibited healed TB lesions on chest radiographs before TNF antagonist use despite having no history of TB treatment. Of these, 2 had negative LTBI screening results and did not receive LTBI treatment, although most guidelines recommend LTBI treatment in such patients^4–6, 16^. The most frequent disease requiring TNF antagonist therapy was RA (36.9%), followed by AS (24.8%), Crohn’s disease (CD) (20.1%), ulcerative colitis (UC) (11.4%), and “others” (6.8%). The LTBI group had more RA and AS patients than the no-LTBI group, and the no-LTBI group had more inflammatory bowel disease patients than the LTBI group. Underlying comorbidities did not differ significantly between the two groups. With regard to TNF antagonists, infliximab (29.6%), adalimumab (27.9%), golimumab (7.3%), and etanercept (11.7%) were administered, and 23.5% of the patients were treated with ≥2 TNF antagonists. Most patients (678; 96.6%) used other immunosuppressive drugs with the TNF antagonists. The median duration of TNF antagonist use was 15.5 months (range 0.5–114.8) and the median follow-up duration was 32.1 months (0.5–131.3). The LTBI group had a longer median duration of TNF antagonist use and a longer follow-up period than the no-LTBI group (TNF antagonists use duration, 18.6 *vs*. 12.7 months; follow-up duration, 32.6 *vs*. 31.8 months, respectively) (Table 1).

### Diagnosis and treatment of LTBI

Of the 702 patients, 168 (23.9%) had positive IGRA results. Of the total of 512 patients who underwent TSTs, 165 (32.2%) yielded positive results (data not shown). A total of 255 patients were diagnosed with LTBI before TNF antagonist use. Of these, 78 had both IGRA and TST positive results, whereas 177 were either IGRA positive (90 patients) or TST positive (87 patients) (Fig. 1). Among the patients who were IGRA negative but TST positive, 9 did not undergo LTBI treatment because their treating physician thought the results could be false positives due to Bacillus Calmette–Guérin (BCG) vaccination. Thus, with the exception of these 9 patients, 246 of the 255 (96.5%) received LTBI treatment. Table 2 shows the LTBI treatment regimens and outcomes. Two hundred and nineteen patients (89.0%) were treated with 9 months of daily isoniazid (9H), and 27 patients (11.0%) were treated with 3 months of daily isoniazid plus rifampicin (3HR). Median duration from LTBI treatment to starting TNF antagonist was 24 days (IQR, 21–35) and there was no statistical difference between 9H and 3HR groups (24 *vs*. 28 days). Of the total of 219 9H LTBI patients, 186 (84.9%) completed the treatment. Of those that did not, 9 stopped using the drug due to side effects, 20 were lost to follow-up, and 4 stopped the LTBI treatment because they also stopped TNF antagonist therapy. Of the patients treated with 3HR, only 1 (3.7%) stopped the LTBI treatment due to side effects. Five patients developed active TB during the follow-up period, and all of them were in the 9H treatment group.

**Table 2:** Treatment regimens and outcomes for LTBI in 246 patients.

	LTBI treatment regimen	3 HR	Total	*P*
	9H			
Number (%)	219/246 (89.0)	27/246 (11.0)	246	
Completed treatment*, n (%)	186/219 (84.9)	26/27 (96.3)	212 (86.2)	0.142
Not completed, n (%)	33/219 (15.1)	1/27 (3.7)	34(13.8)	
side effects	9^†^	1^‡^	9	
Dropout	20	0	20	
stop due to TNF antagonist stop	4	0	4	
Duration from LTBI treatment to initiation of TNF antagonist, median (IQR), days	24 (21–35)	28 (14–90)	24 (21–35)	0.110
Active TB occurrence during follow up	5/219 (2.3)	0/27 (0)	5	NS

Data are presented as numbers (percentages) unless otherwise indicated. *Completed Treatment was defined as the ingestion of >80% of all prescribed medications within 43 weeks for 9H or within 16 weeks for 3HR. ^†^Five hepatitis, two active TB during LTBI treatment, one skin rash and one mood change. ^‡^One skin rash. LTBI, latent tuberculosis infection; H, isoniazid; HR, isoniazid plus rifampicin; TNF, tumor necrosis factor; TB, tuberculosis; NS, not significant.

### Clinical characteristics of 11 active TB patients

Table 3 shows clinical characteristics of the 11 active TB patients during or after TNF antagonist therapy. Five developed active TB during or after LTBI treatment, and of these, 3 exhibited isoniazid-resistant drug sensitivity. Six developed TB despite baseline negative LTBI screening results. Among the 11 active TB cases, 3 were extrapulmonary TB and the others were pulmonary and military TB. The mean duration of TNF antagonist use in the active TB patients was 9.9 months (range 0.7–31.5), and the mean duration from TNF antagonist initiation to TB onset was 20.7 months (range 2.0–73.2).

**Table 3:** Clinical characteristics of 11 active TB patients during or after TNF antagonists therapy.

Case	1	2	3	4	5	6	7	8	9	10	11
Age/sex	47/M	50/F	55/M	27/M	57/F	20/F	79/F	55/M	56/F	33/M	49/M
Underlying disease	CD	RA	CD	AS	RA	CD	RA	CD	RA	CD	RA
Anti-TNF agent used	Ada + INF	Ada	INF	Gol	Eta + Ada	INF	Eta + Ada	INF	Ada + Eta	INF	INF
Baseline chest x-ray	normal	normal	healed TB^*^	normal	healed TB^*^	normal	normal	normal	normal	normal	normal
Baseline TST induration, mm	0	29	15	10	11.5	ND	0	ND	0	0	0
Baseline IGRA	Positive	Positive	Positive	Positive	Positive	Negative	Negative	Negative	Negative	Negative	Negative
LTBI Tx	Yes	Yes	Yes	Yes	Yes	No	No	No	No	No	No
LTBI Tx regimen	H	H	H	H	H	—	—	—	—	—	—
Duration from LTBI Tx to initiation of TNF antagonist, days	21	31	21	43	34	—	—	—	—	—	—
LTBI Tx completion	completed	completed	interrupted	interrupted	completed	—	—	—	—	—	—
Duration of anti-TNF therapy, months	10.7	13.1	1.4	1	23	20.6	31.5	3.3	3.3	1.2	0.7
Time to TB after TNF antagonist use, months	30.9	34.9	2.9	3.4	23.5	21.9	73.2	4.2	28.8	2.4	2
Type of TB	EPTB	PTB	PTB	EPTB	PTB	PTB	PTB	PTB	PTB	EPTB	PTB
TB diagnosis method	culture(+) LN Bx	culture(+) sputum	culture(+) sputum	culture(+) LN Bx	culture(+) sputum	culture(+) sputum	culture(+) sputum	culture(+) sputum	culture(+) sputum	culture(+) peritoneal	culture(+) sputum
TB drug sensitivity	all S	INH-R	INH-R	INH-R	all S	all S	all S	all S	all S	all S	all S
TB Tx outcomes	completed	completed	completed	completed	death	completed	death	completed	completed	completed	completed

*Healed TB in chest radiography was defined as apical fibronodular lesions, calcified lymph nodes, calcified solitary nodules, and pleural thickening. TB, tuberculosis; TNF, tumor necrosis factor; CD, crohn’s disease; RA, rheumatoid arthritis; AS, ankylosing spondylitis; Ada, adalimumab; INF, infliximab; Gol, golimumab; Eta, etanercept; TST, tuberculin skin test; ND, not done; IGRA, interferon gamma release assay; LTBI Tx, latent tuberculosis infection treatment; H, isoniazid; EPTB, extrapulmonary TB; PTB, pulmonary TB; LN Bx, lymph node biopsy; all S, all sensitive; INH-R, isoniazid resistant.

### Incidence ratio of active TB after TNF antagonist therapy

Table 4 shows the observed numbers and SIR of active TB. In the LTBI group, 5/255 (1.96%) patients developed active TB associated with TNF antagonist therapy during the study period (897.60 patient-years of follow-up). Thus, the incidence of TB in TNF antagonist therapy with LTBI was estimated to be 557.04/100,000 patient-years. After adjustment for age and sex, the SIR was 6.01 (95% CI 1.94–14.04). In the baseline negative LTBI group before TNF antagonist therapy, 6/447 (1.34%) patients developed active TB during the study period (1336.53 patient-years follow-up). The incidence of TB in TNF antagonist therapy with initial negative LTBI was estimated to be 448.92/100,000 patient-years, and the SIR was 5.14 (95% CI 1.88–11.18).

**Table 4:** The observed numbers and standardized incidence ratio of patients with active TB.

LTBI (+)	Patients	Follow up duration (y)	Patient-years	Observed	Expected*	SIR	95% CI
Male	134	3.19	427.46	3	0.38	7.89	1.28–23.04
Female	121	3.89	470.69	2	0.32	6.16	0.69–22.23
Age (years)							
20–29	14	2.83	39.62	1	0.03		
30–39	40	2.97	118.8	0	0.08		
40–49	70	4.14	289.8	1	0.19		
50–59	62	3.39	210.18	3	0.18		
60–69	46	3.81	175.26	0	0.21		
70–79	23	2.82	64.86	0	0.15		
≥80	0	0	0	0	0		
Overall	255	3.52	897.60	5	0.71	6.01	1.94–14.04
**No LTBI**	**Patients**	**Follow up duration (y)**	**Patient-years**	**Observed**	Expected*	**SIR**	**95% CI**
Male	222	2.98	661.56	3	0.59	5.10	1.02–14.89
Female	225	3.00	675.00	3	0.47	6.44	1.29–18.82
Age (years)							
20–29	112	2.68	300.16	1	0.25		
30–39	121	2.87	347.27	1	0.23		
40–49	93	3.24	301.32	1	0.20		
50–59	58	3.41	197.78	2	0.17		
60–69	41	2.8	114.8	0	0.14		
70–79	18	3.17	57.06	1	0.13		
≥80	4	4.69	18.76	0	0.06		
Overall	447	2.99	1336.53	6	1.06	5.14	1.88–11.18

*To estimate ‘expected TB’ the sex and age specific TB patient notifications in 2011 were extrapolated from Korea centers for disease control and prevention. TB,tuberculosis; SIR, standardized incidence ratio; CI, confidence interval.

## Discussion

Previous studies have reported that TNF antagonist therapy increased the risk of active TB by approximately 1.6–25.1 times^1–3, 17–19^. Recently, Navarra *et al*.^11^ suggested that the risk of developing TB with TNF antagonist therapy is higher in Asia than in Western Europe and North America, due to a higher burden of TB incidence. In the current study, the SIR of active TB in patients treated with TNF antagonists was 6.01 in the LTBI group and 5.14 in the initial negative LTBI group. Our findings are comparable with those of a study conducted in another tertiary referral hospital in Korea^20^. However, according to a recent large cohort multicenter study in 873 inflammatory bowel disease patients receiving TNF antagonists in Korea, the adjusted SIR was 41.7 compared with that of a age and sex matched sample of the general population^21^. Possible reasons for this discrepancy are differences in LTBI screening rates, screening methods, and rates of LTBI chemoprophylaxis. Most patients in the current study (808/823, 98.2%) were screened for LTBI prior to TNF antagonist therapy, and the screening methods used were IGRA with or without TST in accordance with Korean guidelines in immunocompromised patients. Furthermore, 246/255 (96.5%) patients diagnosed with LTBI received chemoprophylaxis. Conversely, the aforementioned large multicenter study^21^ reported that the overall LTBI screening rate was 81.6%, furthermore, 12.9% of patients were screened by only the TST, and 86.3% of LTBI patients received chemoprophylaxis. TNF antagonists are increasingly used to treat various inflammatory diseases in Korea. However, LTBI screening and its treatment before TNF antagonist therapy have not been performed completely depending on the physician and hospital, in the real world. The differences of standardized incidence of TB ratio between current study and the multicenter study described above^21^ emphasized the importance of proper screening prior to TNF antagonist therapy and LTBI treatment to reduce the incidence of active TB development.

There is insufficient evidence of the effectiveness of the different LTBI chemoprophylaxis regimens. In most countries, 9 months of treatment with isoniazid (9H) is currently recommended to prevent TB in LTBI, however, alternative regimens such as 3 months of treatment with isoniazid plus rifampicin (3HR) or 4 months of treatment with rifampicin (4R) have also been considered acceptable^2^. A study conducted in 2008, 92.6% of active TB patients in South Korea were sensitive to all anti-TB drugs and 7.4% of patients were resistant to one or more anti-TB drugs^22^ Another study in 2004 reported that 5% of TB patients were resistant to isoniazid at first treatment and 7% among retreatment patients^23^. Considering the lower rate of drug resistant active TB in South Korea, Korea guidelines still recommend 9H LTBI treatment regimen as a first line therapy. However, 4R and 3HR regimens are also accepted. In current study, 219/246 (89%) patients were treated with the 9H regimen, whereas 27/246 (11%) were treated with the 3HR regimen. However, the chemoprophylaxis completion rates were 84.9% for 9H and 96.3% for 3HR. During follow-up, 5 patients developed TB despite chemoprophylaxis of LTBI and all of these patients were treated with the 9H regimen, though notably this difference in active TB incidence rate according to regimen was not statistically significant, due to the relatively small percentage of patients treated with 3HR. Three out of 5 patients were resistant to isoniazid. Possible reasons for those results were H resistant LTBI or developing H resistant active TB after 9H chemoprophylaxis. We cannot confirm the exact sequence of incidence, however, we have to consider special situation like exposure to H resistant TB patients before deciding LTBI regimen. Several studies have suggested that the 3HR or 4R regimens seem to be superior to the 9H regimen due to the relatively shorter course of treatment, high completion rate, and acceptable rate of adverse drug reactions^13, 20, 24^. That suggestion seems reasonable, especially in patients with low compliance, or in cases where there is an urgent need for TNF antagonists with a short induction period of chemoprophylaxis and a high risk of reactivating LTBI.

In this current study, 2 patients revealed healed TB lesion on chest x-ray with negative TST and IGRA result (Table 1). Although this 2 patients didn’t develop active TB without LTBI treatment, previous studies reported that healed TB lesion without anti-TB treatment history increased risk of developing active TB about 6–19 times^25, 26^. Therefore many guidelines recommend LTBI treatment before anti-TNF therapy even though negative IGRA or TST result if patient’s chest x-ray had healed TB lesion without treatment history^4–6^. Among initial LTBI positive group, only 3.5% (9/255) patients have TB scars on x-ray (Table 1), but among active TB patients after LTBI treatment, 2 out of 5 (40%) patients have TB scars (Table 3). UK guideline recommends completion of LTBI treatment before anti-TNF therapy in patients with TB scars due to high risk of developing TB^5^. A study conducted in South Korea suggested 3HR seemed to be superior to the 9H regimen especially in patients with high risk of reactivating LTBI^20^. In respect that patients with TB scars have increased risk of developing TB, it seems reasonable that 3HR prophylactic regimen is more appropriate rather than 9H mono-therapy.

In a previous study, the majority of cases of TB related to TNF antagonist therapy occurred close to the time of treatment initiation^27^. In the present study, of a total of 11 active TB patients, 5 (45.5%) developed active TB within 6 months of the initiation of TNF antagonist therapy, and 6 (54.5%) developed TB 20 months after the initiation of TNF antagonist therapy. This biphasic occurrence of active TB development has also been reported in another study^28^. Possible reasons for these results include reactivation of LTBI in the early period of TNF antagonist therapy, and possible *de novo* infection with TB in the late period of long-term TNF therapy. In the current study, 6 patients developed active TB despite baseline negative LTBI. Among these patients, 3 developed TB within 6 months. Considering the short interval between the initiation of TNF antagonist therapy and the onset of active TB, the emergence of active TB in these patients may have been associated with false negative screening results^29^. With regard to overcoming this problem, several studies have suggested that IGRA and TST combination screening method is a safer, and more appropriate strategy for detecting LTBI in immune-mediated inflammatory disease, especially in high TB incidence^30–32^. The Korean Guidelines for Tuberculosis^4^ recommend either IGRA only or IGRA and TST in combination for screening for LTBI in immunocompromised patients. However, detecting LTBI with both IGRA and TST combination methods in patients at high risk of LTBI reactivation such as those undergoing TNF antagonist therapy may be worth considering. Of the 11 patients with newly developed TB in the current study, 6 developed TB 20 months after the initiation of TNF antagonist therapy. Among these, 3 developed TB after LTBI treatment and 3 developed TB even though they showed negative results in LTBI screening. Although they could not recall close contact with active TB patients, it is possible that long-term TNF antagonist therapy may predispose patients to both *de novo* TB infection and reactivation of LTBI. To detect *de novo* TB infection, some experts have suggested that repeated testing for LTBI using IGRA may be considered in patients at ongoing risk of TB exposure (*i.e*., via travel, work, or by way of living in a high TB incidence country)^33^.

The current study has several limitations. It was conducted at only a single, tertiary hospital; hence the findings are not representative of all patients undergoing TNF antagonist therapy in Korea. It is difficult to assess the risk factors for TB development, due to the relatively small number of active TB cases. LTBI and no LTBI groups are not exactly matched in clinical characteristics especially the duration of TNF antagonist use which could affect developing TB risk and acquiring LTBI. SIR methodology in the TB incidence also has a limitation because of not being corrected for the underlying disease. Furthermore, due to the retrospective nature of the study, accurate BCG vaccination histories and histories of close contact with active TB patients could not be obtained. Notably, the study also has several strengths. It provided information about baseline LTBI status before TNF antagonist therapy and the difference in TB incidence according to baseline screening results. Further predictive risk stratification methods need to be investigated via multicenter, well-designed studies.

## Conclusion

In the current study, the incidence of TB in patients treated with TNF antagonists increased even after LTBI treatment, and also in patients who exhibited baseline negative LTBI screening results. During the first several months of TNF antagonist therapy, short regular follow-ups and close monitoring of signs and symptoms compatible with active TB is important due to the high risk of LTBI reactivation. Clinicians should also be aware of the risk of the development of active TB after several years, even after LTBI treatment and despite baseline negative LTBI screening results. Therefore, regular monitoring and serial tests need to be considered during long-term TNF antagonist therapy, to detect *de novo* TB infection, especially in intermediate to high TB burden countries.
